# Correlating Chemical Fingerprint to Sensory Evaluation: A Four-Vintage Study (2017–2020) of Xinomavro Red Wine

**DOI:** 10.3390/foods15091592

**Published:** 2026-05-04

**Authors:** Artemis Toulaki, Dimitrios Kalompatsios, Martha Mantiniotou, Vassilis Athanasiadis, Kosmas Roufas, Stavros I. Lalas

**Affiliations:** 1 Department of Agriculture, University of Western Macedonia, Terma Kontopoulou, 53100 Florina, Greece; info@wineconsulting.gr; 2 Department of Food Science & Nutrition, University of Thessaly, Terma N. Temponera Street, 43100 Karditsa, Greece; dkalompatsios@uth.gr (D.K.); mmantiniotou@uth.gr (M.M.); vaathanasiadis@uth.gr (V.A.); k.roufas@pdm.gov.gr (K.R.)

**Keywords:** oak barrel aging, vintage examination, sensory evaluation, total polyphenols, volatile compounds, color coordinates, bioactive molecules, correlation analyses

## Abstract

Understanding how chemical composition, phenolic profile, volatile compounds and sensory attributes evolve during wine aging is essential for optimizing quality in premium red wines. This study investigated four consecutive vintages (2017–2020) of Xinomavro red wine (PDO Amyndeo), each produced under identical viticultural and enological conditions and aged for either 12 or 24 months in French oak barrels. Comprehensive chemical, phenolic, colorimetric and volatile analyses were combined with sensory evaluation and multivariate statistics to elucidate vintage- and aging-driven differences. Wines aged for 24 months exhibited higher phenolic richness and antioxidant capacity, with the 2020–24 m sample showing the highest total polyphenols (4230 mg GAE/L) and FRAP values. Sensory analysis revealed clear differentiation among vintages, with younger wines expressing red fruit and tomato leaf aromas, while older vintages displayed dried fruit, caramel, spice and oak-derived notes. PCA demonstrated that PC1 captured phenolic and antioxidant variation, PC2 reflected chromatic intensity and residual sugars, and PC3 represented spicy/vegetal and acidity-related attributes. Multivariate correlation analysis confirmed strong associations between phenolic indices, volatile compounds, and sensory descriptors. Overall, the integration of chemical fingerprinting, volatile profiling and sensory evaluation provides valuable insights into how vintage and barrel aging shape the complexity and quality of Xinomavro wines.

## 1. Introduction

Since ancient times, optimization of viticulture and enological techniques have led to several grape (*Vitis vinifera*) varieties with distinct sensory attributes, including aromatic profile, taste, terroir, and color [1]. Greece produces more than 200 million liters of wine annually and ranks among the top 20 wine-producing countries worldwide, with approximately 10% of its production classified as Protected Designation of Origin (PDO). Xinomavro is the second most planted red variety in Greece, representing nearly one-third of total red wine production [2]. This iconic cultivar is predominantly grown in northern Greece, particularly in the PDO regions of Goumenissa, Naoussa, Rapsani and Amyndeo [3]. Xinomavro grapes and wines are characterized by moderate color intensity, high concentrations of stable anthocyanins—especially malvidin derivatives—and elevated tannin levels [4], resulting in naturally astringent wines with remarkable aging potential [5]. This variety is also highly sensitive to climatic variation [6], responsive to canopy conditions, and classified as late-ripening [7]. Recent studies have also highlighted the strong influence of vintage-specific climatic conditions on grape composition and wine quality, particularly in late-ripening cultivars such as Xinomavro.

High-quality wines typically originate from regions where vine growth is moderated by environmental constraints, while viticultural practices further influence grape composition and final wine quality [8]. Aging is a key determinant of wine sensory properties, and French oak barrels remain the industry standard for maturation [9]. Oak wood contains lignins, tannins, lactones and vanillin [10], which release volatile compounds such as lactones, esters, aldehydes and higher alcohols during barrel contact [11]. These compounds contribute floral, smoky, spicy, caramel and woody notes, depending on barrel origin and toasting level [12].

Wine aroma and flavor are shaped by multiple factors, including terroir, vineyard management and aging conditions, all of which influence the concentration of volatile compounds [13]. Consequently, analytical techniques capable of accurately characterizing wine aroma are essential. The adsorption of odorous compounds using headspace solid-phase microextraction (HS-SPME) and the identification process using gas chromatography–mass spectrometry (GC-MS) are widely used for volatile profiling due to their sensitivity and reproducibility [14]. Sensory evaluation, particularly quantitative descriptive analysis, complements instrumental data by capturing human perception of aroma, taste and mouthfeel [15]. Together, analytical and sensory approaches provide a comprehensive assessment of wine quality.

Xinomavro red wines are considered a hallmark of Greek viticulture and have been the subject of several studies focusing on the ageing process of Xinomavro red wine and their olfactory and bioactive composition [16,17,18,19]. In addition, vintage variation is a known determinant wherein different climatological conditions highly affect the composition of grapes in ripening stage [20,21]. However, limited research has examined how barrel aging influences the integrated chemical, phenolic, volatile and sensory profile of Xinomavro across multiple vintages. This represents a significant knowledge gap, particularly given the increasing consumer demand for red wines with distinctive aromatic complexity. Therefore, in this study, we investigated four consecutive vintages (2017–2020) of Xinomavro PDO Amyndeo, each aged for either one or two years in French oak barrels, to evaluate how aging duration shapes their chemical fingerprint, volatile composition and sensory attributes. The findings aim to support optimized winemaking strategies and deepen understanding of aging-driven quality evolution in Xinomavro wines.

## 2. Materials and Methods

### 2.1. Reagents, Solvents, and Winemaking Materials

Folin–Ciocalteu reagent and gallic acid were both purchased from Panreac Co. (Barcelona, Spain). Formic acid (98%), sodium carbonate, and vanillin were bought from Penta (Prague, Czech Republic). Chloride salts (iron (III) chloride, aluminum chloride, and sodium chloride), acids (L-ascorbic acid, hydrochloric acid), and 2,4,6-tris(2-pyridyl)-*s*-triazine (TPTZ), along with 2,2-diphenyl-1-picrylhydrazyl (DPPH radical), alcohols (methanol, ethanol, and 2-octanol) and all chemicals of HPLC grade were bought from Sigma-Aldrich (Darmstadt, Germany). Acetonitrile was from Labkem (Barcelona, Spain).

Winemaking yeast strain Vitilevure^®^ DV10 was obtained from Martin Viallatte (Magenta, France). Purified pectinolytic enzymes LAFASE™ HE GRAND CRU derived from *Aspergillus niger* were bought from Laffort (Bordeaux, France). Fermentation activator and yeast nutrients Nutriferm Start and Nutriferm Advance were obtained from Enartis (Trecate, Novara, Italy).

### 2.2. Xinomavro Wine Samples

#### 2.2.1. Viticultural Details

**Figure 1:**
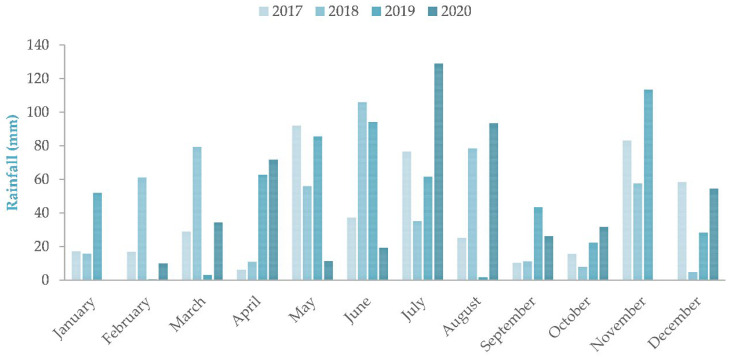
Average rainfall (mm) of the Amyndeo region of Florina, Greece across vintages 2017–2020.

**Figure 2:**
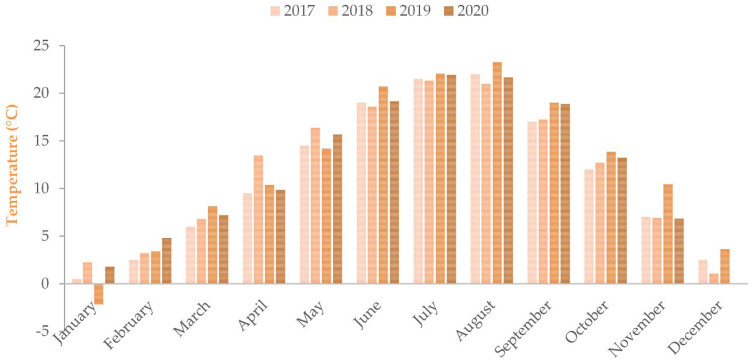
Average temperature (°C) of the Amyndeo region of Florina, Greece across vintages 2017–2020.

The vineyard used in this study is located in Agios Panteleimon, Amyndeo, Greece (40°44′4″ N, 21°44′22″ E), at an altitude of 570 m with a 5–8% slope. Google Earth (version 9.185.0.0) was used for geographic coordinates. It covers 1.2 ha and is planted exclusively with Xinomavro (*Vitis vinifera* L.), established in 2007–2008 in a traditional cup-shaped (goblet) training system. Figure 1 and Figure 2 illustrate the climatic data of the Amyndeo region in Florina, Greece, as presented by the Hellenic National Meteorological Service [22]. The vineyard is non-irrigated and managed consistently across all years to ensure uniformity. The average yield is approximately 600 kg per acre. Annual cultivation practices begin with winter pruning in early February, followed by the application of manure or iron-rich fertilizers to address nutrient deficiencies. A cultivator is used for soil management, and at budbreak, a wettable sulfur treatment is applied to prevent eriophyid mite infestation. Depending on weather conditions, sulfur applications are repeated every 20 days for approximately 40 days. During flowering, sulfur powder is applied to protect against powdery mildew, with a potential second application after lateral shoot development and before bunch closure. After veraison, copper formulations are used to maintain foliage health. In seasons with late-September rainfall, an organic botrytis control preparation may be applied. Harvest typically occurs between 5 and 13 September. To obtain representative grape samples, berries were collected randomly from multiple vines and canopy positions, ensuring exposure to varying light conditions.

#### 2.2.2. Vinification Procedure

Winemaking was carried out under identical conditions for all vintages to ensure experimental consistency. Grapes were processed in a 3-ton stainless steel fermenter. Upon reception of the must, 60 g/ton of sodium metabisulfite was added. Cold maceration was performed for 3 days at 10 °C. Subsequently, the temperature was gradually increased, reaching a maximum of 25 °C on the 10th day. Cap management was conducted through pump-overs (remontage) at decreasing frequency: six pump-overs per day during the first seven days, followed by three pump-overs per day until the 10th day. The wine was separated from the pomace (devatting) on the 11th day. The alcoholic fermentation lasted a total of 17 days, and fermentation kinetics were monitored daily through density measurements.

The must was inoculated on the fourth day with 150 g/ton of the commercial yeast strain Vitilevure^®^ DV10 (Martin Viallatte). The initial yeast-assimilable nitrogen (YAN) concentration ranged from 54 to 67 mg/L. Nutrient supplementation consisted of Nutriferm Start (200 g/ton, Enartis) at the initiation of alcoholic fermentation together with the yeast, followed by Nutriferm Advance (200 g/ton, Enartis) after a 30-unit decrease in must density. A pectinolytic enzyme preparation (Lafase™ HE Grand Cru, Laffort; 20 g/ton) was added at crushing to enhance color and phenolic extraction.

Malolactic fermentation occurred spontaneously, relying exclusively on indigenous lactic acid bacteria, without commercial inoculation. The process was not completed in certain vintages, as also verified by residual malic acid levels. After alcoholic fermentation, an additional 60 g/ton of sodium metabisulfite was added. Free SO_2_ levels were monitored monthly and adjusted to maintain a target of 30 mg/L. Vintage-specific free and total SO_2_ values are presented in Table 1. Following vinification, wines were transferred to medium-toast French oak barrels, comprising 10% new, 40% one-year-old, and 50% two-year-old barrels. Aging was conducted for either 12 or 24 months depending on the treatment. All wines were bottled using natural cork closures (49 mm × 24 mm) and stored under controlled conditions (14 °C, 70% humidity) until analysis.

#### 2.2.3. Xinomavro Wines

All wines used in this study were produced from Xinomavro grapes cultivated in the same vineyard and vinified under identical enological conditions, as described above. For each vintage (2017–2020), wines were aged either for 12 months or for a total of 24 months in medium-toast French oak barrels (10% new, 40% one-year-old, and 50% two-year-old). After maturation, all wines were bottled with natural cork closures (49 mm × 24 mm) and stored under controlled conditions (14 °C, 70% humidity) until analysis. All samples were analyzed in December 2025 immediately after opening. Technological parameters were determined as per the official procedures of the International Organization of Vine and Wine (OIV) [23].

### 2.3. Bioactive Compounds Measurement

#### 2.3.1. Total Anthocyanins

To conduct all spectrophotometric analyses and determinations, we used a double-beam UV-1900i spectrophotometer from Shimadzu (Kyoto, Japan). The total anthocyanin content (TAC) of wine samples was measured using an established methodology [24]. The concentration of total anthocyanins (*C_TA_*) was determined utilizing the widely known Bee–Lambert law. We expressed the results as cyanidin-3-*O*-glucoside equivalents (CyE) per L of wine. The equation incorporated the absorbance (*A*) that we recorded as well as the dilution factor (*F_D_*) of each wine sample. Data concerning cyanidin-3-*O*-glucoside involved its molecular weight (*MW*) of 449.2 and molar absorptivity (*E*) of 26,900.

(1) $$C_{\mathrm{TA}}\;(\mathrm{mg}\;\mathrm{CyE}/L)=\;\frac{A\;\times \;\mathrm{MW}\;\times \;F_{D}}{E\;\times {\;10}^{3}}$$

#### 2.3.2. Total Tannins

The total tannin content (TTC) of wines was examined using the vanillin assay, as previously described [24]. This process involves the reaction of condensed tannins with vanillin, resulting in the formation of a stable, red-pigmented compound. The results are presented as mg of catechin equivalents (CtE) per L of wine, utilizing a calibration curve which is shown in Table A1.

#### 2.3.3. Total Flavonoids

The total flavonoid content (TFC) of wine samples was quantified using an established methodology [24]. The spectrophotometric absorbance of a yellowish generated complex was the principle of this method. TFC was quantified using a calibration curve, as detailed in Table A1. We expressed the results as mg of rutin equivalents (RtE) per L of wine.

#### 2.3.4. Total Polyphenols

The total polyphenol content (TPC) of each wine sample was ascertained using an adjusted procedure [24] of the established Folin–Ciocalteu assay. The absorbance at 740 nm of blueish complex was measured, and the TPC was determined using a calibration curve which is visible in Table A1. The findings are presented as mg of gallic acid equivalents (GAE) per L of wine.

#### 2.3.5. Polyphenolic Compounds Quantification

We obtained high-performance liquid chromatography (HPLC) apparatus from Shimadzu (Kyoto, Japan). In particular, the CBM-20A compartment was equipped with diode array detector (DAD, model SPD-M20A). The compounds that were identified were separated using a non-polar chromatographic column by Phenomenex Inc. (Torrance, CA, USA). The column had analytical dimensions that allowed for adequate separation of compounds. Analysis of individual polyphenolic compounds was conducted using HPLC and a previously conducted methodology [24]. A non-polar column model Luna C(18)2 with analytical dimensions (100 Å, 5 μm, 4.6 mm × 250 mm) from Phenomenex Inc. (Torrance, CA, USA) was employed. The column had a stable temperature throughout the procedure (40 °C). The injection volume was set at 20 μL, and the autosampler had a stable temperature of 4 °C. A constant flow rate (1.0 mL/min) was employed by using two solvent mixtures. The first mixture (A) consisted of 0.5% *w*/*v* formic acid in water, whereas mixture B consisted of 0.5% *w*/*v* formic acid in acetonitrile. The chromatographic programme included an initial 95% A which gradually reduced at 60% in a 10 min span and to 50% after another 10 min. Finally, a further reduction to 30% A was completed in 10 min, and the specific solvent composition (i.e., 30% A, 70% B) was kept constant for 40 min, totaling a 70 min procedure. We were able to quantify the identified compounds by employing calibration curves ranging from 0 to 250 µg/mL, as also presented in Table A2. We express the results in μg per mL of wine. However, quantification of anthocyanins was conducted utilizing cyanidin-3-glucoside chloride, with the results expressed as cyanidin equivalents.

### 2.4. In Vitro Antioxidant Activity

#### 2.4.1. Ion-Reducing Activity

The ion-reducing capacity of wine samples was assessed using the ferric-reducing antioxidant power (FRAP) assay, as previously described [24]. The principle of this method is based on the redox reaction (under acidic conditions) of the ferric (III) to (II) ion to form a stable blueish complex following electron transfer from antioxidant molecules. The observed ion-reducing capacity of each wine samples was evaluated utilizing a calibration curve as illustrated in Table A1. We expressed the results as mmol of ascorbic acid equivalents (AAE) per L of wine.

#### 2.4.2. Radical Scavenging Activity

The wine samples were also tested for their antioxidant activity by evaluating radical scavenging capability using the DPPH^•^ probe method [24]. The principle of this assay lies in the decolorization of a purple DPPH^•^ solution, as the radical is stabilized by hydrogen atom donation by antioxidant molecules. We evaluated the antiradical activity by utilizing an ascorbic acid calibration curve (Table A1). We express the results as mmol of AAE per L of wine, using the same expression for antioxidant activity assays.

### 2.5. Sensory Attributes and Chemical Characterization of Xinomavro Wines

#### 2.5.1. Volatile Compound (VC) Identification Through HS-SPME/GC-MS

Volatile compounds were determined using a previously described HS-SPME protocol [24]. A DVB/CAR/PDMS fiber over-coated with PTFE (Supelco, Bellefonte, PA, USA) was conditioned at 270 °C for 30 min prior to use. For each wine sample, we mixed 3 g of sodium chloride in 10 mL of wine into a 25 mL glass vial, along with 2 mg/L of 2-octanol which served as the internal standard. An equilibration step was followed, wherein the mixture was kept at a constant temperature (40 °C) in an oil bath and a stirring hotplate for 10 min. After sealing the vial, we placed the holder and exposed the fiber for the microextraction process. The glass vial was under constant stirring at 250 rounds per minute in an oil bath. The stirring process lasted for 40 min. Right after, the fiber was inserted into GC-MS equipment.

The gas chromatograph was equipped with a J&W DB-1 non-polar column from Agilent Technologies (Santa Clara, CA, USA). Helium was used as the carrier gas (1.5 mL/min, constant flow). The injector had a constant temperature (240 °C) in splitless mode. The oven programme was set at 40 °C for 5 min, then increased to 140 °C by 2 °C/min, and then rose to 240 °C by 20 °C/min. The programme lasted for 70 min. Mass spectrometry apparatus had specific parameters including an ion source 230 °C, quadrupole 150 °C, and electron ionization energy set at 69.9 eV within a scan range of 29–450 *m*/*z*. Solvent delay was set at 0.01 min.

Compounds were identified using mass spectral libraries with ≥80% match quality. Regarding the quantification of obtained peaks, we employed normalization procedure, excluding correction factors. We finally expressed the results as mg of 2-octanol equivalents per L of wine.

#### 2.5.2. Sensory Evaluation of Wines

Aroma and taste analyses of all Xinomavro samples were conducted by 10 trained panelists, all of whom had prior experience in wine sensory evaluation and participated in a short calibration session before the analysis. Each wine was assessed independently. Constant volumes of wine (20 mL) were served in standard wine-tasting glasses according to ISO 3591 [25], which were covered with watch glasses to minimize volatile loss. Evaluations were carried out in the sensory analysis chambers of the Department of Food Science and Nutrition (University of Thessaly, Karditsa, Greece) [26] under controlled conditions (uniform lighting, absence of distracting odors or noise, constant room temperature (20 °C), and individual booths). Panelists sniffed and tasted each wine sample (*n* = 8) and generated individual sensory terms. A 0–5 quantitative intensity scale (0 = not perceived, 5 = very intense) was used to evaluate all sensory attributes. The age range of assessors was 25–55 years. All wine samples were evaluated in triplicate, and the same 10 assessors participated in all sessions to ensure consistency.

### 2.6. Color Analysis

We measured every wine sample with a colorimeter from The Tintometer Ltd. (Amesbury, UK), model Lovibond CAM-System 500. We identified the CIE 1976 L* a* b* color coordinates of Xinomavro wine samples that define brightness (L*), redness (a*), and blueness (b*). Both the hue and saturation of each color were determined using the chroma (C*) parameter and hue angle ($h_{ab}^{o}$), using the following formulae:

(2) $$C_{ab}^{*}=\sqrt{{{(a}^{*})}^{2}+{{(b}^{*})}^{2}}$$

(3) $$h_{ab}^{o}=\arctan\left(\frac{b^{*}}{a^{*}}\right)$$

### 2.7. Statistical Processing

To conduct statistical processing of our results, we employed JMP^®^ Pro 16 software (SAS Institute, Cary, NC, USA). All chemical, phenolic, colorimetric and volatile measurements were performed in triplicate, and results are expressed as mean ± standard deviation. To evaluate the effects of vintage and aging duration, we applied both a οne-way ANOVA (for comparisons within each vintage–aging combination) and a two-way ANOVA (vintage × aging duration) for all chemical and phenolic variables. Tukey’s HSD test (*p* < 0.05) was used for post hoc comparisons. Sensory data were collected using a 0–5 quantitative intensity scale and therefore treated as continuous variables.

To explore relationships among analytical and sensory attributes, Pearson correlation coefficients were calculated for all continuous variables. A Principal Component Analysis (PCA) was employed using the correlation matrix to identify major sources of variation across samples. Variable clustering was used to group correlated parameters into chemometrically coherent clusters. A Multivariate Correlation Analysis (MCA) was also conducted to visualize the correlation structure of the dataset. All statistical analyses were performed using JMP^®^ Pro 16 (SAS Institute, Cary, NC, USA).

## 3. Results and Discussion

### 3.1. Physicochemical Profiling of Xinomavro Wines

The physicochemical properties and oenological parameters of Xinomavro wines differed not only between vintages but also between the 24-month “Reserve” and 12-month control wines, as illustrated in Table 2. Alcohol content was significantly higher (*p* < 0.05) in the more recent vintages, indicating consistent fermentation efficiency. This trend was also supported by density values, which showed no significant variation across vintages. Volatile acidity remained low (0.41–0.63 g acetic acid/L), confirming microbiological stability in all samples.

To address vintage-dependent differences in spontaneous malolactic fermentation, we provide a summary of key fermentation indicators (density, alcohol, malic acid, lactic acid, pH, total acidity) for each vintage in Table 2. These data confirm that vinification conditions were consistent across years, while natural biological variability explains the observed differences in MLF progression.

Total acidity was highest in the 2017 vintage (7.3 g/L), followed by a gradual decrease in subsequent vintages, likely due to incomplete malolactic fermentation, as supported by residual malic acid levels. Malolactic fermentation occurred spontaneously at 20 °C, resulting in variable conversion efficiency among vintages. pH values ranged from 3.14 to 3.49, showing only minor differences, likely attributed to residual malic, lactic, and tartaric acid concentrations.

The obtained results were consistent with our previous study, which involved several Xinomavro cultivars (including Amyndeo) from 2022 vintage [18]. Similar values to oenological parameters were observed in the study from Kyraleou et al. [27]. For instance, similar pH values (~3.4), alcohol content (~15.5%), and total acidity (~6.5 g/L) were measured in Xinomavro wines. The two-way ANOVA (vintage × aging duration) confirmed the same statistical trends observed in the one-way ANOVA, with no contradictory interaction effects (Table A3).

### 3.2. Bioactivity Determination of Xinomavro Wines

#### 3.2.1. Bioactive Compound and Antioxidant Capacity Evaluation

The quantification of bioactive compounds revealed statistically significant differences (*p* < 0.05) across vintages (Table 3). Total polyphenols ranged from 2451.8 to 4230.17 mg GAE/L, with the 2020–24 m sample exhibiting nearly double the concentration of the older vintages. The highest concentration was measured in the 2018 vintage with 12-month aging. It was observed that 2018 had moderate rainfall and temperatures (specifically a “dry” September), resulting in an optimal growing season; this could possibly explain the huge polyphenol yield. The sharp decline in the “reserve” vintage could be due to the lower stability of polyphenols. A similar pattern was observed for total flavonoids (156.39–248.03 mg RtE/L).

In contrast, total tannins and total pigments showed inverse trends. Total tannins ranged from 113.61 to 202.16 mg CtE/L, with the highest value in 2018–12 m and the lowest in 2017–24 m. Total pigments (anthocyanins) ranged from 60.94 to 78.48 mg CyE/L, with the highest value in 2019–12 m. This variability likely reflects vintage-dependent climatic effects, influencing anthocyanin biosynthesis and degradation [28,29,30]. Although Xinomavro is rich in malvidin-3-O-glucoside, anthocyanins were expressed as cyanidin-3-O-glucoside equivalents for comparison purposes [27,31].

It was observed that our results were superior compared to Tekos et al. [32] who evaluated the bioactivities of Xinomavro Cv. Naoussa and measured ~1600 mg GAE/L. In addition, the previous study from Kyraleou et al. [27] supported this evidence, as the authors measured 1820–2380 mg GAE/L and 22.16–34.51 mg/L of total anthocyanins. These findings are consistent with previous reports showing that climatic variability across vintages significantly affects phenolic maturity, color stability, and volatile composition in red wines. These phenolic differences are also reflected in the chromatic parameters, as color intensity, hue evolution and CIE coordinates are strongly driven by anthocyanin stability and the formation of polymeric pigments during aging.

We observed that antioxidant capacity assays increased throughout the vintages; FRAP values gradually rose from 27.32 (2017 vintage) to 54.47 mmol AAE/L wine (2020 vintage), whereas DPPH was elevated from 16.99 to 27.33 mmol AAE/L wine through the same vintages. The obtained results aligned and mirrored the pattern from total polyphenols, further supporting that the latter vintage was the most potent in terms of bioactivities. The two-way ANOVA (vintage × aging duration) supported these trends, showing significant main effects and interactions for most phenolic and antioxidant variables (Table A3).

#### 3.2.2. Polyphenolic Profile

**Figure 3:**
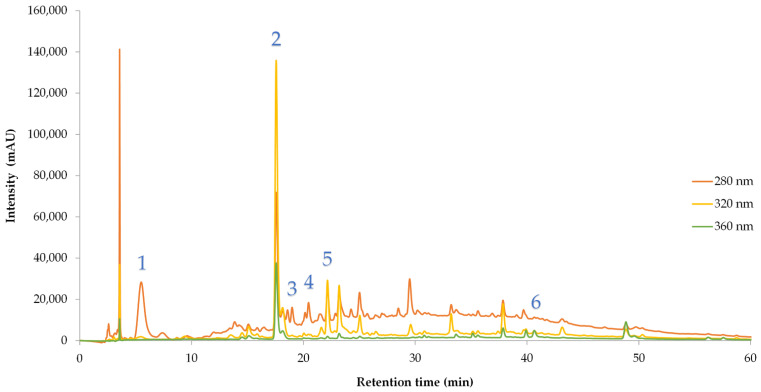
Representative HPLC chromatogram of the Xinomavro wine sample (2019—24 months aging), recorded at 280 nm (orange), 320 nm (yellow) and 360 nm (green). Peaks correspond to (1) gallic acid, (2) neochlorogenic acid, (3) pelargonin chloride, (4) catechin, (5) chlorogenic acid, and (6) myricetin. Retention times and UV–Vis spectra matched authenticated standards.

Individual polyphenols (Table 4, Figure 3) ranged from 56.98 to 332.47 mg/L. A general decrease in monomeric phenols (gallic acid, neochlorogenic acid) was observed with extended aging, consistent with oxidation and polymerization reactions [33]. A notable exception was the 2019 vintage, which showed a sharp increase in catechin after 24 months, suggesting tannin depolymerization or vintage-specific extraction dynamics [34]. Overall, the 2019 and 2020 vintages exhibited higher phenolic concentrations than 2017–2018, reinforcing the influence of climatic conditions and fruit maturity on phenolic development. Similar phenolic profiles have been reported in recent studies [35,36].

### 3.3. Chromatic Parameters

The chromatic characteristics of Xinomavro wines (Table 5) showed limited variation. Lightness (L*) exhibited no significant differences (*p* > 0.05). Redness (a*) values were relatively low (12.5–18.4) but statistically different (*p* < 0.05). Yellow–blue values (b*) were also low (2.0–6.9), with the highest in 2020–24 m. Chroma (C*), indicating color saturation, was higher in the more recent vintages, likely due to shorter bottle aging and reduced pigment degradation. The subtle color differences among vintages may reflect anthocyanin composition, particularly cyanidin derivatives. We observed that the 2018 vintage had stable and low hue but only modest increases in redness and saturation during aging. On the other hand, the 2020 vintage (with high rainfall in July and August) showed a significant increase in both redness and saturation, which suggests that wet vintages could still develop excellent color during barrel aging through polymeric pigment formation. Compared to Karimali et al. [36], who reported L* = 21.76, a* = 45.16, b* = 29.18, our wines exhibited darker, less saturated colors, consistent with their higher phenolic content.

### 3.4. Volatile Compound Profiling

Volatile compound profiling revealed a complex and vintage-dependent aromatic evolution (Table 6). Total VOCs ranged from 2.05 to 12.67 mg/L, increasing with wine maturity. These compounds were also identified in previous investigations regarding Xinomavro red wine [16,19,36]. Older vintages (2017–2018) were dominated by isoamyl alcohol, reaching concentrations nearly ten-fold higher than other VOCs, imparting a solvent-like fusel character [37]. However, these vintages showed a decline in total VOCs between 12 and 24 months, suggesting rapid degradation or transformation of early-stage volatiles [38].

In contrast, the more recent vintages (2019–2020) exhibited a positive maturation trajectory, characterized by increasing levels of diethyl succinate and ethyl lactate, esters associated with fruity, floral and creamy notes formed via slow esterification reactions [39]. The behavior of 2-phenylethanol, a key rose-like aromatic [40], further highlighted vintage-specific differences: it increased during the second year of aging in 2019 and 2020 but decreased in 2017 and 2018, suggesting differences in redox stability and aromatic retention. Overall, older vintages showed high initial fusel alcohol dominance, whereas recent vintages developed more balanced, ester-driven aromatic complexity, possibly reflecting improved viticultural or enological practices.

### 3.5. Sensory Evaluation

The sensory assessment revealed clear differences among vintages and aging durations, fully aligned with the chemical and phenolic composition of the wines. Wines from younger vintages (2020) exhibited aromas of fresh red fruits, tomato leaf and olive, accompanied by more assertive and youthful tannins, consistent with their higher tannin content and lower polymerization degree. The 24-month-aged samples showed greater aromatic depth, with spicy notes, enhanced structure and better integrated mouthfeel, reflecting the impact of extended barrel maturation.

The 2019 vintage presented a markedly different profile, characterized by nutmeg, caramel, dried red fruits and fig jam, in agreement with its higher residual sugar and volatile acidity. The palate was lively yet softer, with acidity requiring time to integrate—a finding supported by the higher total acidity and lower pH observed analytically.

The Reserve wines consistently demonstrated richer, more complex aromatic profiles, dominated by spice, dried tomato, red pepper and subtle vanilla from oak integration. Their tannins were described as velvety and well rounded, matching the higher polymeric tannin content and the significant Vintage × Aging interaction observed in the phenolic analysis.

Older vintages such as 2017 displayed high acidity, dried fruit complexity and sweet spice, with a long and elegant finish. The Reserve counterpart showed enhanced aromatic complexity, including sweet smoke and dried tomato, with exceptionally smooth tannins—characteristics consistent with the elevated flavonoid levels and pigment stability measured analytically.

Overall, the sensory results corroborate the chemical and phenolic data, confirming that vintage strongly shapes aromatic and structural attributes, while extended aging enhances complexity, softens tannins, and integrates oak-derived notes. Table 7 summarizes the key aroma, palate and structural attributes identified by the tasting panel.

**Figure 4:**
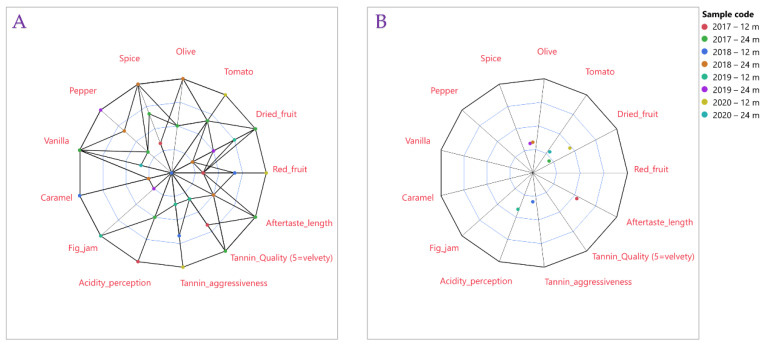
Sensory visualization of Xinomavro wines using spider (**A**) and radar (**B**) plots.

The sensory profiles were visualized using both spider and radar plots (Figure 4). The spider plot highlights the magnitude of each sensory attribute, while the radar plot provides an integrated view of the overall sensory footprint. Together, they illustrate clear differences among vintages and aging durations, particularly in red fruit intensity, dried fruit notes, spice attributes, and tannin quality.

Correlation analysis revealed strong associations between sensory attributes, indicating coherent aromatic and structural patterns across vintages. Tannin quality was strongly negatively correlated with tannin aggressiveness (r = −0.918), confirming the internal consistency of the sensory evaluation. Vanilla intensity, associated with oak aging, showed a strong positive correlation with both pepper (r = 0.874) and tannin smoothness (r = 0.866), highlighting the role of barrel maturation in modulating mouthfeel and aromatic complexity. The Mediterranean savory profile (olive–tomato) also appeared as a unified sensory dimension (r = 0.909).

### 3.6. Correlation Analyses

#### 3.6.1. Principal Component Analysis (PCA)

**Figure 5:**
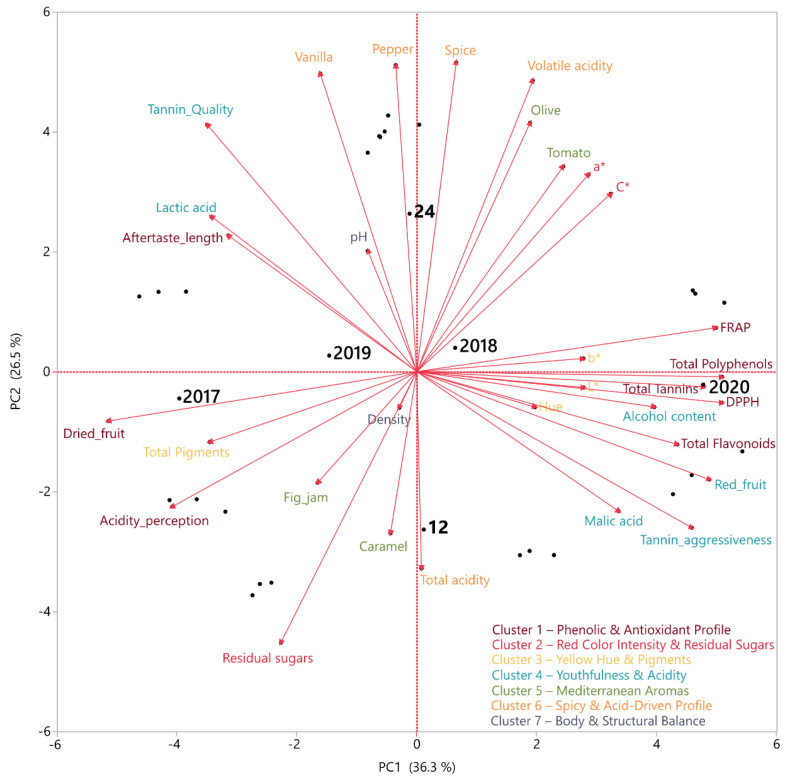
PCA biplot (PC1 × PC2) of chemical, phenolic, color, and sensory attributes of Xinomavro wines.

In Figure 5, the PCA reveals that PC1 (36.3%) was associated with the phenolic and antioxidant profile of the samples, PC2 (26.5%) reflected color intensity and residual sugars, and PC3 (13.5%) captured the spicy/vegetal character and acidity. Variable clustering further confirmed the co-occurrence of phenolic and sensory maturity indicators, as well as the distinct grouping of color parameters.

Overall, the PCA explained 76% of the total variance, indicating clear differentiation among samples according to vintage and aging duration. Older wines (2017–2018) were characterized by dried fruit, oxidative and phenolic attributes, whereas younger wines (2019–2020) were associated with red fruit freshness, higher acidity, and spicy notes. Variable clusters highlighted coherent chemometric groupings, including phenolic, chromatic, spicy/oak-related, and Mediterranean aromatic dimensions.

#### 3.6.2. Multivariate Correlation Analysis (MCA)

**Figure 6:**
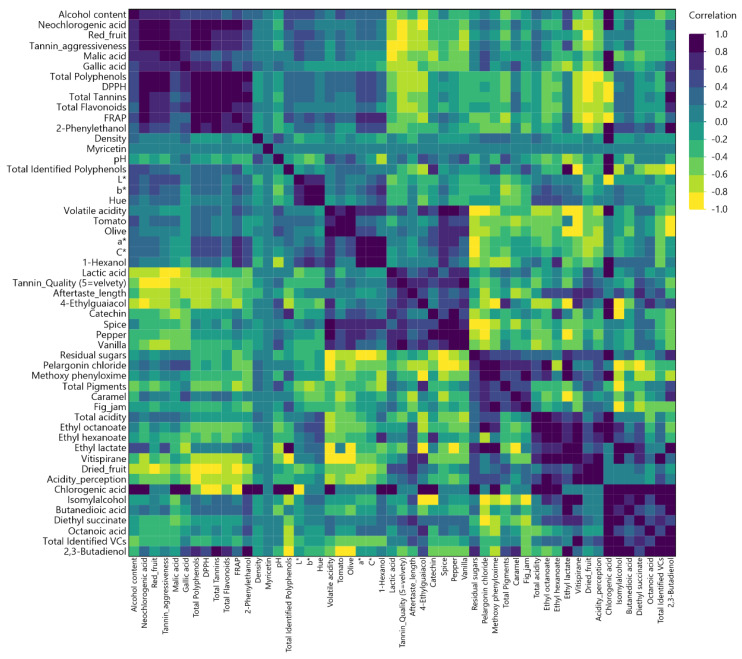
Multivariate correlation map of chemical, phenolic, volatile, and sensory attributes of Xinomavro wines.

In Figure 6, MCA revealed strong associations among chemical, phenolic, volatile and sensory variables. Total polyphenols, total tannins, FRAP, and DPPH were highly correlated (r > 0.90), confirming the coherence of the phenolic–antioxidant system. Malic and lactic acid showed a strong negative correlation (r = −0.92), reflecting the progression and variability of malolactic fermentation across vintages.

Sensory descriptors associated with maturity (dried fruit, aftertaste length) correlated positively with phenolic indices, whereas red fruit intensity correlated with malic acid, indicating a link between freshness and acidity. Spicy and oak-related attributes (pepper, spice, vanilla) were positively associated with volatile acidity, suggesting an oxidative and barrel-derived aromatic dimension.

### 3.7. Integrated Interpretation

The combined evaluation of chemical composition, phenolic profile, chromatic attributes, volatile compounds, and sensory perception reveals a coherent and multidimensional picture of how vintage and barrel aging shape the identity of Xinomavro wines. Across all analytical layers, a consistent pattern emerged: younger vintages (2019–2020) were characterized by higher acidity, fresher red fruit aromas, elevated malic acid, and increased ester formation, whereas older vintages (2017–2018) displayed greater phenolic maturity, dried fruit complexity, spicy notes and smoother tannins.

The phenolic and antioxidant data demonstrated that wines with higher total polyphenols, flavonoids, and tannins also exhibited stronger antioxidant capacity (FRAP, DPPH), confirming the tight coupling between phenolic richness and redox potential. These findings aligned with the PCA results, where PC1 captured phenolic density and antioxidant strength, clearly separating vintages according to their maturity and aging trajectory.

Volatile compound profiling further reinforced this differentiation. Older vintages showed initial dominance of fusel alcohols, followed by a decline during extended aging, whereas recent vintages accumulated fermentation-derived esters such as diethyl succinate and ethyl lactate, contributing to fruitier and more balanced aromatic profiles. The behavior of 2-phenylethanol and other key volatiles reflected vintage-specific redox stability, consistent with the MCA correlations linking volatile acidity, oak-derived notes and spicy attributes.

Colorimetric data, although less variable, supported the chemical findings: wines with higher pigment stability and phenolic content exhibited greater chroma and more saturated hues, particularly in the Reserve samples. These chromatic differences corresponded with sensory descriptors related to visual intensity and perceived maturity.

Sensory evaluation provided the final layer of integration. Attributes such as dried fruit, spice, vanilla, aftertaste length and tannin smoothness were strongly associated with phenolic indices and oak-related volatiles, while red fruit intensity and freshness correlated with malic acid and acidity. The MCA confirmed these relationships, revealing coherent aromatic and structural dimensions that reflect both chemical composition and human perception.

Overall, the integrated chemometric interpretation demonstrates that vintage exerts the strongest influence on the chemical and sensory architecture of Xinomavro wines, while extended barrel aging enhances complexity, promotes phenolic evolution, stabilizes color, and deepens aromatic structure. The convergence of analytical and sensory data highlights the distinctive aging potential of Xinomavro and underscores the importance of coordinated chemical–sensory evaluation in understanding its quality evolution.

## 4. Conclusions

This study demonstrated that both vintage variation and French-oak barrel aging play a decisive role in shaping the chemical, phenolic, chromatic, volatile and sensory profile of Xinomavro cv. Amyndeo wines. Extended aging for 24 months consistently enhanced aromatic complexity, phenolic maturity and tannin smoothness, while also improving antioxidant capacity and stabilizing color attributes. Among the vintages examined, the 2020 wines—particularly after prolonged aging—exhibited the highest concentrations of total polyphenols and antioxidant activity, accompanied by vivid color and intense, well-integrated aromatic expression. The results highlight that vintage exerts a strong influence on phenolic development, volatile composition, and sensory character, suggesting that climatic conditions may modulate flavonoid and tannin biosynthesis. Future studies incorporating detailed climatic data could further clarify these relationships and provide predictive insights into polyphenolic maturation under varying environmental conditions. From a practical standpoint, the findings indicate that extending barrel aging to 24 months can serve as a strategic enological approach to enhancing the structural depth, aromatic richness, and oxidative stability of Xinomavro wines. Such regulated aging practices can elevate the sensory profile toward a more complex and consumer-preferred style, effectively bridging the gap between traditional production and contemporary market expectations. Overall, this work underscores the remarkable aging potential of Xinomavro and provides a comprehensive framework for optimizing its quality through integrated chemical and sensory evaluation.

## Figures and Tables

**Table 1:** Different composition of sulphur dioxide (SO_2_) during vinification process of each vintage.

Xinomavro Wine Vintage	Free SO_2_ (mg/L)	Total SO_2_ (mg/L)
2020	21 ± 1.2 ^b^	36 ± 1.9 ^c^
2019	20 ± 0.9 ^b^	52 ± 2.6 ^b^
2018	32 ± 1.6 ^a^	65 ± 2.9 ^a^
2017	18 ± 0.7 ^b^	34 ± 1.3 ^c^

Displayed values as their mean values along with standard deviation. Several superscript letters (a–c) in the same column reveal major statistical differences (*p* < 0.05) among different vintages.

**Table 2:** Basic oenological parameters of Xinomavro wine samples by vintage and aging duration.

Sample Code	Alcohol (% *v*/*v*)	Density (g/mL)	Residual Sugars (g/L)	Volatile Acidity (g/L)	Total Acidity (g/L)	pH	Malic Acid (g/L)	Lactic Acid (g/L)
2020—12 m	14.3 ± 0.2 ^a^	0.993 ± 0.005 ^a^	0.5 ± 0.01 ^d^	0.52 ± 0.01 ^c,d^	6.8 ± 0.2 ^b^	3.33 ± 0.05 ^a,b^	2.1 ± 0.02 ^a^	0.1 ± 0 ^f^
2020—24 m	14.1 ± 0.37 ^a^	0.989 ± 0.002 ^a^	0.2 ± 0 ^e^	0.58 ± 0.01 ^b^	6.6 ± 0.07 ^b^	3.14 ± 0.09 ^b^	1.2 ± 0.01 ^b^	0.3 ± 0.01 ^e^
2019—12 m	13.3 ± 0.21 ^b^	0.993 ± 0.004 ^a^	0.9 ± 0.02 ^a^	0.43 ± 0.01 ^e^	6.8 ± 0.11 ^b^	3.25 ± 0.05 ^b^	1.1 ± 0.01 ^c^	0.6 ± 0.01 ^c^
2019—24 m	13.2 ± 0.29 ^b^	0.992 ± 0.003 ^a^	0.1 ± 0 ^f^	0.61 ± 0.01 ^a,b^	6.5 ± 0.07 ^b^	3.31 ± 0.1 ^a,b^	0.4 ± 0.01 ^f^	1.1 ± 0.03 ^a^
2018—12 m	13.2 ± 0.13 ^b^	0.992 ± 0.002 ^a^	0.8 ± 0.02 ^b^	0.49 ± 0.01 ^d^	6.5 ± 0.16 ^b^	3.29 ± 0.04 ^b^	1.1 ± 0.03 ^c^	0.5 ± 0.01 ^d^
2018—24 m	13.1 ± 0.29 ^b^	0.992 ± 0.005 ^a^	0.1 ± 0 ^f^	0.63 ± 0.02 ^a^	6.1 ± 0.13 ^c^	3.49 ± 0.08 ^a^	1.1 ± 0.03 ^c^	0.6 ± 0.01 ^c^
2017—12 m	13.1 ± 0.24 ^b^	0.991 ± 0.002 ^a^	0.7 ± 0.02 ^c^	0.41 ± 0 ^e^	7.3 ± 0.11 ^a^	3.22 ± 0.04 ^b^	0.9 ± 0.03 ^d^	0.6 ± 0.01 ^c^
2017—24 m	13.2 ± 0.36 ^b^	0.991 ± 0.005 ^a^	0.8 ± 0.01 ^b^	0.53 ± 0.02 ^c^	6.1 ± 0.14 ^c^	3.32 ± 0.07 ^a,b^	0.8 ± 0.02 ^e^	0.7 ± 0.01 ^b^

Displayed values as their mean values along with standard deviation. Several superscript letters (a–f) in the same column reveal major statistical differences (*p* < 0.05) among different vintages.

**Table 3:** Bioactive composition and antioxidant capacity of Xinomavro wine samples by vintage and aging duration.

Sample Code	Total Polyphenols (mg GAE/L)	Total Flavonoids (mg RtE/L)	Total Tannins (mg CtE/L)	Total Pigments (mg CyE/L)	FRAP (mmol AAE/L)	DPPH (mmol AAE/L)
2020—12 m	4047.15 ± 51.17 ^b^	221.28 ± 5.22 ^b^	172.57 ± 5.59 ^b^	64.58 ± 1.55 ^d,e^	45.51 ± 0.83 ^b^	27.33 ± 0.95 ^a^
2020—24 m	4230.17 ± 39.28 ^a^	206.56 ± 2.61 ^c^	196.29 ± 6.13 ^a^	60.94 ± 1.8 ^e^	54.47 ± 0.35 ^a^	27.01 ± 0.73 ^a^
2019—12 m	2836.62 ± 39.96 ^e^	177.69 ± 5.08 ^d^	130.69 ± 2.1 ^c,d^	78.48 ± 1.38 ^a^	31.54 ± 0.73 ^d^	19.84 ± 0.36 ^c,d^
2019—24 m	3487.71 ± 31.02 ^c^	195.64 ± 1.27 ^c^	160.95 ± 1.94 ^b^	65.52 ± 2.01 ^d,e^	44.14 ± 0.39 ^b^	22.81 ± 0.51 ^b^
2018—12 m	4228.77 ± 26.11 ^a^	248.03 ± 4.19 ^a^	202.16 ± 2.74 ^a^	70.91 ± 0.75 ^b,c^	45.66 ± 0.71 ^b^	27.72 ± 0.96 ^a^
2018—24 m	3093.08 ± 10.68 ^d^	177.8 ± 5.8 ^d^	141.79 ± 2.4 ^c^	74.08 ± 2.57 ^a,b^	33.85 ± 0.3 ^c^	20.51 ± 0.88 ^c^
2017—12 m	2451.8 ± 9.96 ^g^	156.39 ± 2.61 ^e^	119.73 ± 13.54 ^d,e^	67.1 ± 2.1 ^c,d^	27.32 ± 0.26 ^e^	16.99 ± 0.72 ^e^
2017—24 m	2654.44 ± 16.48 ^f^	161.63 ± 3.11 ^e^	113.61 ± 2.33 ^e^	73.39 ± 0.76 ^b^	28.37 ± 0.69 ^e^	17.93 ± 0.33 ^d,e^

Displayed values as their mean values along with standard deviation. Several superscript letters (a–g) in the same column reveal major statistical differences (*p* < 0.05) among different vintages.

**Table 4:** Individual polyphenolic compounds identified by HPLC in Xinomavro wine samples across vintages and aging duration.

Sample Code	Polyphenolic Compound (mg/L)						
	Gallic Acid	Neochlorogenic Acid	Pelargonin Chloride	Catechin	Chlorogenic Acid	Myricetin	Total Identified
2020—12 m	10.81 ± 0.79 ^a^	129.5 ± 6.73 ^a^	122.09 ± 4.4 ^b^	15.49 ± 0.64 ^b^	<LOQ	<LOD	277.89 ± 12.55 ^b^
2020—24 m	7.91 ± 0.21 ^b,c^	112.71 ± 7.66 ^b^	77.46 ± 2.87 ^c^	10.25 ± 0.7 ^b^	<LOQ	<LOD	208.33 ± 11.44 ^c^
2019—12 m	8.43 ± 0.37 ^b^	87.17 ± 4.36 ^c^	165.39 ± 3.31 ^a^	22.13 ± 1.19 ^b^	<LOQ	<LOD	283.12 ± 9.23 ^b^
2019—24 m	6.43 ± 0.47 ^d^	78.64 ± 5.51 ^c^	61.21 ± 1.78 ^d^	186.19 ± 13.22 ^a^	<LOQ	<LOQ	332.47 ± 20.97 ^a^
2018—12 m	11.31 ± 0.33 ^a^	116.95 ± 7.95 ^a,b^	<LOD	14.08 ± 0.72 ^b^	13.13 ± 0.68	<LOD	155.47 ± 9.68 ^d^
2018—24 m	8.83 ± 0.26 ^b^	54.35 ± 2.83 ^d^	<LOD	<LOD	<LOQ	<LOD	63.18 ± 3.09 ^e^
2017—12 m	6.88 ± 0.25 ^c,d^	50.1 ± 1.25 ^d^	<LOD	<LOD	<LOD	<LOD	56.98 ± 1.5 ^e^
2017—24 m	6.65 ± 0.45 ^d^	55.21 ± 2.71 ^d^	87.63 ± 6.57 ^c^	9.47 ± 0.35 ^b^	<LOQ	<LOD	158.96 ± 10.07 ^d^

Values are displayed as mean values along with standard deviation. Different superscript letters (a–e) in the same column reveal major statistical differences (*p* < 0.05) among different vintages.

**Table 5:** Colorimetric parameters (CIELab) of Xinomavro wine samples by vintage and aging duration.

Sample Code	L*	a*	b*	C*	Hue	HEX Code	Color
2020—12 m	37.4 ± 0.2 ^a^	14.2 ± 1.7 ^b^	4.8 ± 2.3 ^a,b^	15.1 ± 2.3 ^b,c,d^	18.2 ± 5.9 ^a^	715051	
2020—24 m	38.5 ± 1.3 ^a^	18.4 ± 0.8 ^a^	6.9 ± 1.8 ^a^	19.7 ± 0.4 ^a^	20.6 ± 5.6 ^a^	7A4F50	
2019—12 m	36 ± 1.4 ^a^	14.2 ± 0.5 ^b^	2.2 ± 0.4 ^b^	14.4 ± 0.5 ^d^	8.9 ± 1.4 ^a^	6C4C52	
2019—24 m	35.3 ± 0.4 ^a^	17.1 ± 0.4 ^a^	3.3 ± 1.6 ^a,b^	17.5 ± 0.3 ^a,b^	10.8 ± 5.4 ^a^	6F494E	
2018—12 m	35.6 ± 0.2 ^a^	14.5 ± 0 ^b^	2.2 ± 1.8 ^b^	14.7 ± 0.3 ^c,d^	8.7 ± 6.8 ^a^	6B4B51	
2018—24 m	36.4 ± 0.9 ^a^	17.1 ± 0.9 ^a^	2.7 ± 0.8 ^a,b^	17.3 ± 1 ^a,b,c^	9 ± 2.1 ^a^	714B52	
2017—12 m	36.6 ± 2.3 ^a^	12.9 ± 0.8 ^b^	4.8 ± 2 ^a,b^	13.9 ± 0 ^d^	20.5 ± 8.9 ^a^	6D4E4F	
2017—24 m	35.4 ± 1.3 ^a^	12.7 ± 0.4 ^b^	2 ± 1.3 ^b^	12.9 ± 0.6 ^d^	8.7 ± 5.6 ^a^	684C50	

Displayed values as their mean values along with standard deviation. Several superscript letters (a–d) in the same column reveal major statistical differences (*p* < 0.05) among different vintages.

**Table 6:** Volatile compounds identified and semi-quantified in Xinomavro wines from four vintages using HS-SPME/GC-MS (mg/L).

Compound	CAS Number	RT (min)	2020—12 m	2020—24 m	2019—12 m	2019—24 m	2018—12 m	2018—24 m	2017—12 m	2017—24 m
Isoamyl alcohol	123-51-3	3.151	n.d.	n.d.	0.09 ± 0 ^d^	n.d.	8.94 ± 0.48 ^a^	4.51 ± 0.26 ^c^	9.48 ± 0.64 ^a^	7.03 ± 0.21 ^b^
2,3-Butadienol	513-85-9	4.373	0.08 ± 0 ^c^	0.19 ± 0.01 ^b^	n.d.	0.1 ± 0 ^c^	0.29 ± 0.02 ^a^	n.d.	n.d.	n.d.
Ethyl lactate	97-64-3	4.767	n.d.	0.49 ± 0.01 ^a^	n.d.	n.d.	n.d.	0.21 ± 0.01 ^b^	n.d.	0.51 ± 0.01 ^a^
1-Hexanol	111-27-3	6.98	0.14 ± 0.01 ^c^	0.31 ± 0.01 ^a^	n.d.	0.2 ± 0.01 ^b^	0.14 ± 0 ^c^	n.d.	n.d.	0.14 ± 0 ^c^
Methoxy phenyloxime	1000222-86-6	9.921	0.09 ± 0 ^b,c^	0.05 ± 0 ^e^	0.21 ± 0.01 ^a^	0.08 ± 0 ^c,d^	0.06 ± 0 ^d^	0.03 ± 0 ^f^	0.1 ± 0 ^b^	0.06 ± 0 ^d,e^
Ethyl hexanoate	123-66-0	13.7	0.06 ± 0 ^c,d^	0.08 ± 0 ^b^	0.06 ± 0 ^c^	0.09 ± 0.01 ^b^	0.06 ± 0 ^c^	0.05 ± 0 ^d^	0.18 ± 0.01 ^a^	0.06 ± 0 ^c,d^
2-Phenylethanol	60-12-8	20.178	0.9 ± 0.05 ^b^	1.45 ± 0.03 ^a^	0.62 ± 0.03 ^d^	0.8 ± 0.05 ^b^	1.43 ± 0.07 ^a^	0.88 ± 0.02 ^b^	0.78 ± 0.05 ^b,c^	0.67 ± 0.02 ^c,d^
Diethyl succinate	123-25-1	25.358	1.16 ± 0.05 ^c^	1.41 ± 0.05 ^b^	0.91 ± 0.04 ^d^	1.48 ± 0.09 ^a,b^	1.43 ± 0.09 ^a,b^	1.34 ± 0.07 ^b,c^	1.62 ± 0.07 ^a^	1.48 ± 0.08 ^a,b^
Ethyl octanoate	106-32-1	27.115	0.15 ± 0.01 ^b^	n.d.	n.d.	0.12 ± 0.01 ^c^	0.11 ± 0.01 ^c^	0.11 ± 0.01 ^c^	0.23 ± 0.01 ^a^	0.12 ± 0 ^c^
Octanoic acid	124-07-2	27.985	0.09 ± 0 ^e^	0.28 ± 0.02 ^a^	0.13 ± 0 ^d^	0.13 ± 0.01 ^d^	0.25 ± 0.01 ^b^	0.27 ± 0.01 ^a,b^	0.18 ± 0.01 ^c^	0.26 ± 0.01 ^a,b^
4-Ethylguaiacol	2785-89-9	31.322	0.02 ± 0 ^d^	0.03 ± 0 ^c^	n.d.	0.03 ± 0 ^c^	0.03 ± 0 ^c^	0.05 ± 0 ^a^	n.d.	0.03 ± 0 ^b^
Vitispirane	65416-59-3	31.812	0.02 ± 0 ^c^	n.d.	n.d.	0.02 ± 0 ^c^	n.d.	n.d.	0.04 ± 0 ^a^	0.03 ± 0 ^b^
Butanedioic acid	28024-16-0	41.369	0.05 ± 0 ^e^	0.08 ± 0 ^a,b^	0.04 ± 0 ^e^	0.06 ± 0 ^d^	0.09 ± 0.01 ^a^	0.01 ± 0 ^f^	0.06 ± 0 ^c,d^	0.07 ± 0 ^b,c^
Total Identified VCs			2.74 ± 0.13 ^e^	4.36 ± 0.14 ^d^	2.05 ± 0.1 ^e^	3.1 ± 0.19 ^e^	12.82 ± 0.7 ^a^	7.47 ± 0.39 ^c^	12.67 ± 0.78 ^a^	10.46 ± 0.35 ^b^

Displayed values as their mean values along with standard deviation. Several superscript letters (a–e) in the same column reveal major statistical differences (*p* < 0.05) among different vintages. n.d. means “not detected”.

**Table 7:** Sensory attributes of Xinomavro wines across vintages and aging durations.

Vintage	Aging	Aroma Profile	Palate/Acidity	Tannins	Aftertaste/Overall Impression
2020	12 months	Red fruits, fresh tomato, olive, subtle spicy notes	Lively, balanced, structured	Slightly aggressive due to youth	Well-built, full-bodied
2020	24 months (Reserve)	Deeper, more intense spicy nose with greater aromatic complexity	Rounder palate, better acidity integration	Firm but more mature	Rich, structured finish
2019	12 months	Nutmeg, butter, caramel, dried red fruits, fig jam	Vibrant yet soft, high acidity	Soft tannins	Needs time for full integration
2019	24 months (Reserve)	Intense spicy nose, red pepper, dried tomato, olive, vanilla from oak	Fuller and more mature	Velvety, well-integrated	Sweet vanilla notes, rounded finish
2018	12 months	Sweet caramel notes, red fruit jam	Energetic, full but slightly rustic	More rustic tannins	Assertive finish
2018	24 months (Reserve)	Spicy, peppery, dried tomato, olive, vanilla	More mature and balanced	Velvety tannins	Sweet oak-derived notes, smooth finish
2017	12 months	Dried fruits, sweet spices, chestnut	Soft, velvety, high acidity	Soft tannins	Very long and elegant aftertaste
2017	24 months (Reserve)	Sweet smoke, dried tomato, aromatic spices	Balanced, mature palate	Exceptionally smooth	Harmonious, satisfying finish

## Data Availability

The original contributions presented in the study are included in the article. Further inquiries can be directed to the corresponding author.

## Abbreviations

The following abbreviations are used in this manuscript:

*A*	Absorbance
AAE	Ascorbic Acid Equivalents
CAR	Carboxen
CtE	Catechin Equivalents
CyE	Cyanidin 3-*O*-Glucoside Equivalents
DPPH	2,2-Diphenyl-1-picrylhydrazyl
DVB	Divinylbenzene
*E*	Molecular Absorption
F-C	Folin–Ciocalteu
*F_D_*	Dilution Factor
FRAP	Ferric-Reducing Antioxidant Power
GAE	Gallic Acid Equivalents
GC–MS	Gas Chromatography–Mass Spectrometry
HPLC	High-Performance Liquid Chromatography
HS-SPME	Headspace–Solid-Phase Microextraction
*m*/*z*	Mass-to-Charge Ratio
MCA	Multivariate Correlation Analysis
MSD	Mass Selective Detector
MvE	Malvidin Equivalents
MW	Molecular Weight
OIV	International Organization of Vine and Wine
PCA	Principal Component Analysis
PDMS	Polydimethylsiloxane
PDO	Protected Designation of Origin
PTFE	Polytetrafluoroethylene
RtE	Rutin Equivalents
TAC	Total Anthocyanin Content
TFC	Total Flavonoid Content
TPC	Total Polyphenol Content
TPTZ	2,4,6-Τris(2-pyridyl)-*s*-triazine
TTC	Total Tannin Content
VC	Volatile Compounds
YAN	Yeast Assimilable Nitrogen

## Appendix A

**Table A1:** Linear equation details for each assay.

Assay	Substance	Linear Equation	R^2^	Range	LOD	LOQ
TPC (mg GAE/L)	Gallic acid	*y* = 0.0138*x* − 0.0044	0.9996	10–100	1.75	5.29
TFC (mg RtE/L)	Rutin	*y* = 0.0029*x* + 0.0053	0.9966	30–300	4.42	14.58
TTC (mg CtE/L)	Catechin	*y* = 0.0064*x* + 0.0005	0.9986	10–200	5.61	16.99
FRAP (mmol AAE/L)	Ascorbic acid	*y* = 0.0013*x* − 0.0227	0.9953	0.05–0.5	0.028	0.086
DPPH (mmol AAE/L)	Ascorbic acid	*y* = 0.0625*x* − 0.3165	0.9994	0.1–0.6	0.022	0.066

**Table A2:** Calibration equations, chromatographic characteristics, and detection limits of the polyphenolic standards used for HPLC analysis.

Polyphenolic Compound	Equation	R^2^	RT (min)	UV_max_ (nm)	LOD (mg/L)	LOQ (mg/L)
Gallic acid	*y* = 175,167.40*x* + 105,392.82	0.999	4.72	270	1.56	4.72
Neochlorogenic acid	*y* = 28,213.52*x* + 551.72	0.999	16.50	324	1.74	5.26
Pelargonin chloride	*y* = 1610.01*x* − 2626.92	0.997	18.90	275	2.84	8.61
Catechin	*y* = 11,920.79*x* − 128.19	0.997	20.93	278	2.54	7.71
Chlorogenic acid	*y* = 50,320.40*x* − 23,038.36	0.994	21.95	325	3.67	11.11
Myricetin	*y* = 69,752.64*x* − 57,937.66	0.996	41.15	371	3.20	9.69

**Table A3:** Two-way ANOVA results (Vintage × Aging duration) for chemical, phenolic, and antioxidant variables.

Variable	Effect	F-Value	*p*-Value	Significance
Alcohol	Vintage	21.30	<0.0001	***
	Aging	0.46	0.5074	n.s.
	Vintage × Aging	0.32	0.8083	n.s.
Density	Vintage	0.25	0.8637	n.s.
	Aging	0.68	0.4215	n.s.
	Vintage × Aging	0.39	0.7617	n.s.
Residual sugars	Vintage	1335.97	<0.0001	***
	Aging	8333.01	<0.0001	***
	Vintage × Aging	1951.10	<0.0001	***
Volatile acidity	Vintage	67.40	<0.0001	***
	Aging	644.73	<0.0001	***
	Vintage × Aging	25.79	<0.0001	***
Total acidity	Vintage	13.38	0.0001	***
	Aging	98.88	<0.0001	***
	Vintage × Aging	18.76	<0.0001	***
pH	Vintage	5.63	0.0079	**
	Aging	2.26	0.1519	n.s.
	Vintage × Aging	8.62	0.0012	**
Malic acid	Vintage	2045.23	<0.0001	***
	Aging	2276.27	<0.0001	***
	Vintage × Aging	616.98	<0.0001	***
Lactic acid	Vintage	2637.19	<0.0001	***
	Aging	1805.18	<0.0001	***
	Vintage × Aging	319.43	<0.0001	***
Total Polyphenols (TPC)	Vintage	2811.14	<0.0001	***
	Aging	3.72	0.0716	n.s.
	Vintage × Aging	905.74	<0.0001	***
Total Flavonoids (TFC)	Vintage	251.33	<0.0001	***
	Aging	88.58	<0.0001	***
	Vintage × Aging	140.73	<0.0001	***
Total Tannins (TTC)	Vintage	155.29	<0.0001	***
	Aging	1.68	0.213	n.s.
	Vintage × Aging	73.42	<0.0001	***
Total Anthocyanins (TAC)	Vintage	41.28	<0.0001	***
	Aging	6.45	0.0218	*
	Vintage × Aging	36.86	<0.0001	***
FRAP	Vintage	1489.76	<0.0001	***
	Aging	131.73	<0.0001	***
	Vintage × Aging	527.98	<0.0001	***
DPPH	Vintage	199.08	<0.0001	***
	Aging	9.59	0.0069	**
	Vintage × Aging	56.82	<0.0001	***

n.s. = non-significant (*p* ≥ 0.05); * = *p* < 0.05; ** = *p* < 0.01; *** = *p* < 0.001.
